# Advances in the mechanism of low FODMAP diet in the treatment of irritable bowel syndrome: a review

**DOI:** 10.3389/fnut.2026.1719048

**Published:** 2026-03-12

**Authors:** Binrui Chen, Yawen Zhang, Lijun Du, Jie Jin, Ning Dai

**Affiliations:** 1 Department of Gastroenterology, Sir Run Run Shaw Hospital, School of Medicine, Zhejiang University, Hangzhou, China; 2 Department of Gastroenterology, The Second Affiliated Hospital of Shanghai University (Wenzhou Central Hospital), Wenzhou, Zhejiang, China; 3 The Dingli Clinical College of Wenzhou Medical University, Wenzhou, Zhejiang, China

**Keywords:** gut microbiota, gut-brain axis, immune activation, irritable bowel syndrome, low FODMAP diet, metabolites

## Abstract

Irritable Bowel Syndrome (IBS) is a functional gastrointestinal disorder characterized by abdominal pain associated with changes in stool consistency or bowel movement frequency. It is a highly prevalent chronic relapsing disorder that substantially impairs patients’ quality of life and elevates societal healthcare costs. The low fermentable oligosaccharides, disaccharides, monosaccharides, and polyols (FODMAP) diet has emerged as a cornerstone dietary intervention for IBS owing to its demonstrated efficacy in alleviating symptoms. This article systematically reviews the progress in understanding the mechanism of the low FODMAP diet in the treatment of IBS, covering key dimensions such as reduced intestinal gas production, osmotic regulation, gut microbiota balance, abnormal intestinal fermentation, intestinal inflammatory and immune activation, and improvement in the gut-brain axis function. Additionally, this article reviews predictors of treatment response and outlines future research priorities based on recent evidence.

## 1 Introduction

Irritable Bowel Syndrome (IBS) is a functional gastrointestinal disorder (FGID) characterized by abdominal pain associated with changes in stool consistency or bowel movement frequency. Globally, IBS affects approximately 5–10% of the population and typically follows a chronic relapsing–remitting course (1, 2). The global prevalence of IBS increased by 9.2% from 2006 to 2019 (3). The exact causes of IBS are not fully understood, and common symptom triggers include episodes of acute gastroenteritis, known as post-infectious IBS (4). Genetic factors, abnormal gut-brain interactions, altered intestinal barrier function, changes in gut microbiota, gastrointestinal motility disorders, visceral hypersensitivity, and immune activation abnormalities may also be involved in the pathophysiology of IBS (5). However, for most IBS patients, no single mechanism can fully explain symptom occurrence, making treatment challenging. Most medications targeting the primary symptoms of patients have limited efficacy, and patients often explore alternative strategies (6, 7). Over 80% of IBS patients notice that their symptoms are related to food and often choose to improve their condition through dietary adjustments, such as gluten-free diets and elimination diets based on IgG antibody testing, although these approaches lack strong supporting data (8). Among the available options, the low FODMAP diet has become the most evidence-supported dietary intervention strategy for treating IBS (9).

Currently, the implementation of the low FODMAP diet in clinical practice requires good patient compliance, but the efficacy varies among individuals. Identifying biomarkers through mechanistic research could predict response to the low FODMAP diet, thereby guiding therapy toward likely responders (10). Based on the latest literature, this article systematically reviews the key mechanisms of the low FODMAP diet in treating IBS, aiming to provide a theoretical basis for clinical individualized interventions and promote the transition of treatment toward a precise and personalized model.

## 2 Methodology

A comprehensive literature search was conducted in PubMed and Web of Science using keywords including “low FODMAP diet”, “irritable bowel syndrome”, “gut microbiota”, “short-chain fatty acids”, “mast cells”, “metabolites”, “immune activation” and “gut-brain axis.” The search was restricted to publications from 2005 to 2025 to capture key developments in the field, such as the origin and evolution of the low FODMAP diet. Inclusion criteria specified English-language publications focusing on clinical trials, cohort studies, basic research, or systematic reviews related to the symptoms and underlying mechanisms of IBS. Ultimately, we selected 72 articles for this review.

## 3 Definition of the low FODMAP diet

**Figure 1:**
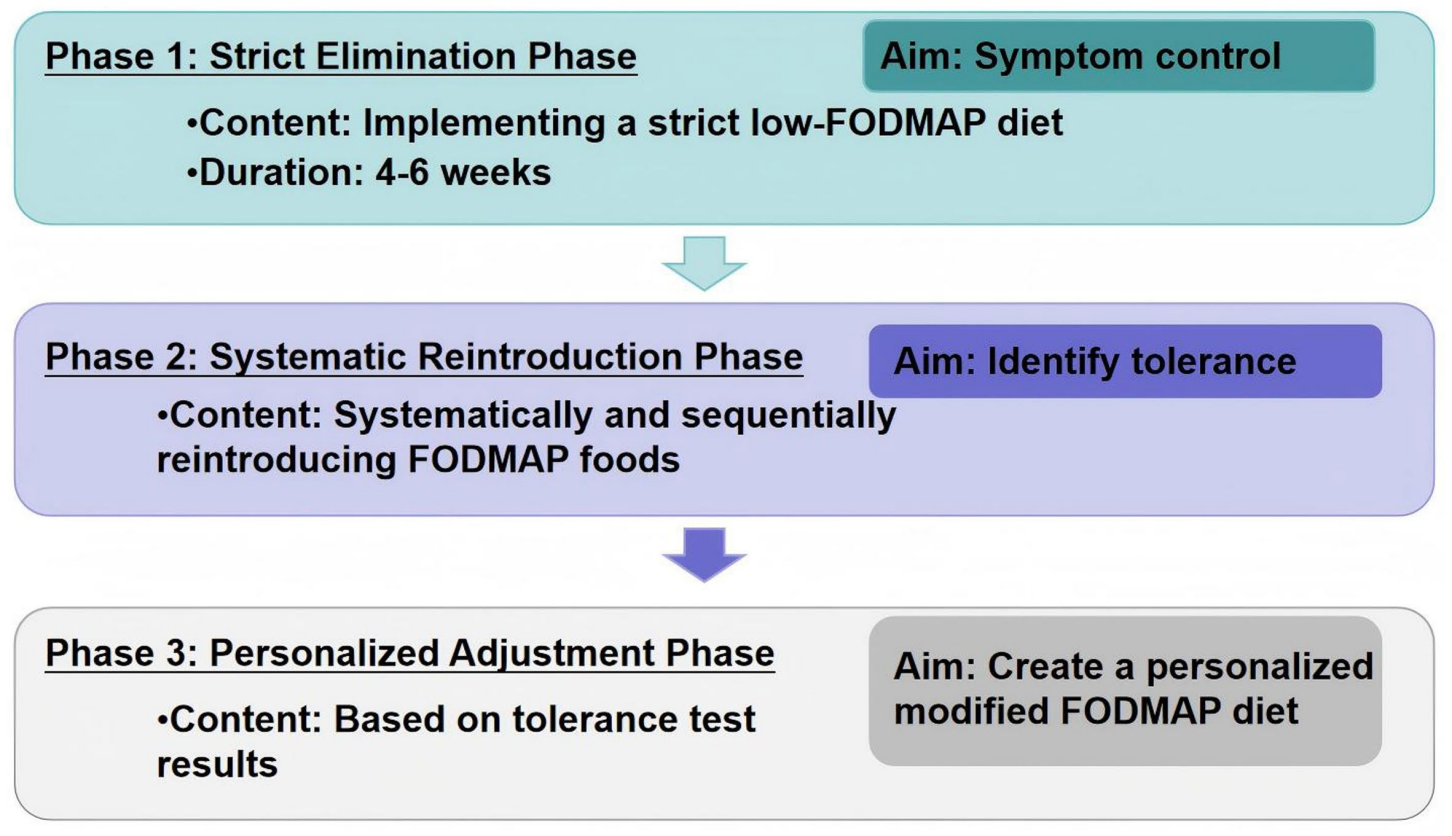
The low FODMAP diet protocol.

FODMAP are a group of easily fermentable short-chain carbohydrates, comprising lactose, fructose, polyols (sorbitol and mannitol), fructans, galacto-oligosaccharides and inulin. These compounds occur in varying concentrations in specific fruits, vegetables, legumes, dairy products, artificial sweeteners, and nuts (11). As a core non-pharmacological intervention for IBS management, the low-FODMAP diet’s efficacy is supported by multiple clinical trials, demonstrating symptom relief in 50–75% of patients, although its mechanisms still require further analysis (8, 12). The low FODMAP diet protocol comprises three phases: the first phase is a strict low FODMAP diet period, ideally lasting 4–6 weeks; the second phase involves reintroducing individual foods to determine tolerance to each; the third phase is to create a personalized modified FODMAP-containing diet strategy based on the individual’s tolerance to different FODMAP foods in the second stage (13) (Figure 1).

## 4 Efficacy profile of the low FODMAP diet

Numerous studies have demonstrated that the low FODMAP diet significantly alleviates IBS symptoms. Multiple systematic reviews with meta-analyses confirm its efficacy in reducing overall symptoms and improving bowel habits (12, 14, 15). Recent umbrella reviews on the low FODMAP diet in IBS present conflicting conclusions: Khalighi Sikaroudi et al. reported broad symptom improvements (e.g., IBS-SSS total score, abdominal pain intensity, abdominal pain frequency, stool consistency and frequency, and quality of life) but no significant effect on belching and urgency of defecation, anxiety and depression, and bloating (16). Zeraattalab-Motlagh et al. reported the low FODMAP diet improves global symptoms, abdominal pain, and stool consistency, and health-related quality of life (QoL) in IBS patients but no notable impact on stool frequency was observed (17). While Bogdanowska-Charkiewicz et al. highlighted limited effects on core gastrointestinal symptoms like abdominal pain and stool frequency, despite benefits in global symptoms and QoL (18). The discrepancies may stem from methodological limitations in Bogdanowska-Charkiewicz’s review, including inconsistent pain assessment scales, lack of IBS subtype stratification, reliance on pre-aggregated meta-analysis data (risking error propagation and overlapping trial weights), and fragile findings (e.g., effect size reversal after excluding a single study). While Bogdanowska-Charkiewicz’s umbrella review highlighted potential placebo or Hawthorne effects as confounders in low-FODMAP diet studies (e.g., patients’ perception and psychological factors contribute to improved QoL or IBS-SSS scores), several high-quality RCTs have rigorously addressed this issue through rigorous experimental design. For instance, double-blind, placebo-controlled trials employing visually and sensorially matched control diets (e.g., FODMAP powder) in rechallenge phases effectively distinguish physiological responses from placebo effects (19). Others utilized strict blinding with standardized food delivery (20) or three-way crossover designs comparing high-FODMAP, high-gluten, and placebo diets, demonstrating that only high-FODMAP diets significantly induced symptoms, thus validating low FODMAP diet’s specificity (21). Shepherd et al. further strengthened this evidence using a quadruple-arm, placebo-controlled rechallenge trial to isolate FODMAP-related physiological effects from placebo effects (22). These studies collectively provide robust evidence that low FODMAP diet’s symptom improvements in IBS extend beyond placebo responses.

### 4.1 Short-term vs. long-term efficacy

The short-term efficacy of the low FODMAP diet has been well established. During the elimination phase (typically 4–6 weeks), strict restriction of FODMAP leads to significant improvements in global IBS symptoms, abdominal pain, and bloating (8, 12). Despite its restrictive nature, emerging studies support the long-term efficacy (e.g., at 12 weeks) of the low-FODMAP diet (23). A systematic review and meta-analysis revealed sustained improvements in all outcomes after FODMAP reintroduction and at the end of follow-up, compared to baseline and real-world PICOS studies (24). Notably, a personalized low FODMAP diet achieved over 50% sustained symptom improvement in IBS patients during follow-up periods of nearly one year (25, 26). Further large-sample researches are necessary to validate the long-term efficacy and safety of the low FODMAP diet.

### 4.2 Comparative efficacy with other dietary interventions

As research on dietary interventions for IBS advances, comparative analyses between the low FODMAP diet and alternative approaches have become a critical focus. In a systematic review and network meta-analysis published in *Gut* in 2022, the low FODMAP diet demonstrated the highest efficacy across all endpoints studied compared to alternative dietary interventions, including the British Dietetic Association (BDA) and National Institute for Health and Care Excellence (NICE) dietary recommendations for individuals with IBS (12). Among dietary interventions for IBS, the low FODMAP diet is supported by the strongest evidence base, although other emerging therapies demonstrate promise and require further investigation (27).

### 4.3 Efficacy differences across IBS subtypes

The efficacy of the low FODMAP diet varies across IBS subtypes, likely due to their distinct pathophysiological features. Most studies focus on IBS with predominant diarrhea (IBS-D) and have consistently demonstrated significant improvements in gastrointestinal symptoms (28). Studies involving IBS with predominant constipation (IBS-C) or mixed bowel habits (IBS-M) have reported comparable symptom reduction, whereas others showed inconsistent outcomes (29, 30), while others showed inconsistent results (31). Future large-sample studies are needed to determine whether the efficacy of the low FODMAP diet remains consistent across different subtypes.

## 5 Core mechanisms of the low FODMAP diet in treating IBS

**Figure 2:**
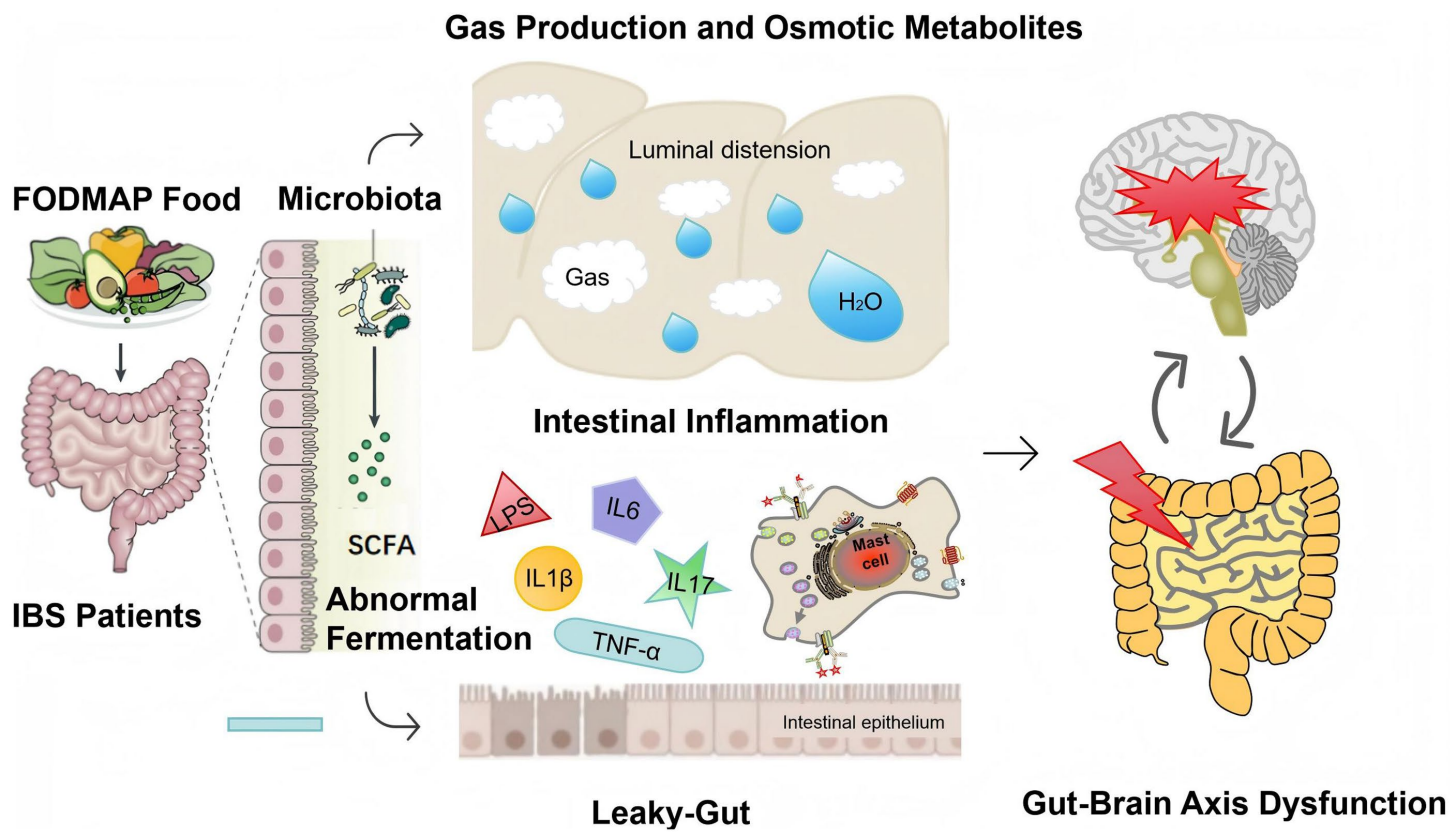
Mechanism of low FODMAP diet in the treatment of irritable bowel syndrome.

A key limitation of most IBS dietary interventions is the incomplete understanding of diet-gut physiology interactions (8). However, accumulating evidence has clarified the multifaceted mechanisms of low FODMAP diet, including reduced gas production, decreased osmotic pressure, reshaped gut microbiota, normalized microbial metabolite profiles, regulated intestinal inflammation, modulated intestinal permeability, and improved gut-brain axis function (Figure 2).

### 5.1 Reduction of intestinal gas production: a primary mechanism for alleviating abdominal pain and bloating

FODMAP remain undigested until reaching the distal intestine, where microbial fermentation produces gases (e.g., hydrogen, methane) causing intestinal distension and gastrointestinal symptoms, especially in patients with visceral hypersensitivity. An intestinal MRI study revealed that although inulin intake produced significantly more colonic gas than glucose in healthy volunteers, it did not induce significant gastrointestinal symptoms, indicating that increased gas production alone is insufficient to cause symptoms (32, 33). Our previous study found that symptoms in Chinese IBS patients after lactose intake were associated with increased hydrogen production and rectal hypersensitivity (34, 35). Studies consistently report that high FODMAP diet increase hydrogen production more markedly in IBS patients with more gastrointestinal symptoms than healthy controls (36), and low FODMAP diet reduce both hydrogen levels and symptom scores (37). Critically, baseline hydrogen production during challenge tests predicts therapeutic response, supporting its role as a biomarker for diet efficacy (38–40) (Supplementary Table 1).

### 5.2 Reducing intestinal content osmolarity: regulating intestinal fluid balance

High FODMAP foods have a high osmolarity due to their molecular structure. Upon entering the gut lumen, they attract water via osmotic effects, leading to fluid accumulation. In IBS patients, this process exacerbates fluid overload in the gut lumen, accelerates motility, induces wall distension, and stimulates visceral sensory nerves, thereby triggering symptoms such as diarrhea and abdominal pain. A randomized, crossover, single-blind intervention trial showed that high FODMAP diet increased the weight, water content, and dry weight of ileal effluent in 10 ileostomy patients, accelerating the transport of water and fermentable substrates to the proximal colon (41). Another study, including healthy volunteers who consumed test meals containing mannitol and glucose and underwent continuous MRI analysis, reported that the small intestinal water content in healthy volunteers after mannitol ingestion was 381 mL, significantly higher than 47 mL observed after glucose ingestion (42). Similarly, fructose consumption in healthy volunteers significantly increased small intestinal water content compared to glucose, whereas inulin consumption did not (32, 33) (Supplementary Table 2). However, the effects of other oligosaccharides (e.g., galacto-oligosaccharides), lactose, and other polyols (e.g., sorbitol) on small intestinal water content remain unclear and require further investigation.

### 5.3 Modulating immune activation: from “microbiomics” to “metabolomics”

Multiple studies have shown that low FODMAP diet may exert its effects by altering IBS patient’s gut microbiota, leading to changes in metabolites and ultimately modulating immune activation (Supplementary Table 3). A recent study from United Kingdom classified baseline IBS patients into pathogenic-like subgroups (enriched in Firmicutes but depleted in Bacteroidetes) and health-like subtypes (microbiota similar to healthy controls) (43). After low FODMAP diet treatment, the pathogenic-like subgroup showed significant symptom improvement and a microbiota shift toward a healthier profile, with increased Bacteroidetes and decreased Firmicutes. Chinese researchers found that low FODMAP diet treatment reduced carbohydrate-fermenting bacteria (e.g., Bifidobacterium and Bacteroides) and decreased glycolytic fermentation activity; patients with higher baseline glycolytic capacity exhibited more severe symptoms and better response (44). In children with IBS, responders at baseline were enriched with bacterial groups possessing stronger glycolytic capacity (e.g., Bacteroidaceae, Ruminococcaceae, and *Faecalibacterium prausnitzii*) (45). Conley et al. reported that IBS patients with baseline enrichment of SCFA fermentation features responded more effectively to low FODMAP treatment, and this group of IBS patients showed a significant reduction in SCFA production after FODMAP restriction (46). Bridgette et al. further identified that elevated baseline fecal propionate and cyclohexanecarboxylic acid could predict responders to low-FODMAP intervention (47). Ameen et al. observed that responders to low FODMAP intervention exhibited a higher abundance of methane and SCFA metabolic pathways in their fecal microbiomes compared to non-responders, suggesting these features may predict treatment efficacy (48). While So et al.’s systematic review found no differences in total or specific fecal SCFA concentrations between low FODMAP completed patients and controls (49), we speculate that subclassifying IBS patients based on response, microbiome, or metabolic subtypes may yield distinct results. This highlights the need for well-designed, high-quality studies, including multi-omics approaches, to further elucidate these metabolic differences.

Although SCFA is considered as prebiotics and its deficiency is increasingly recognized in the pathogenesis of various diseases such as inflammatory bowel disease and colorectal cancer (50), their role in IBS remains unclear. Emerging evidence suggests a potential pro-nociceptive role of fecal SCFA in IBS pathogenesis (51, 52). Isovalerate stimulates enterochromaffin cells to activate sensory neurons through neural signaling (53, 54), and SCFA engages spinal activity via G protein-coupled receptors, stimulating sensory afferent neuron firing (55). Murine studies revealed the IBS group had higher fecal SCFA concentrations and increased colonic transit rates compared to controls (56); in a cohort of IBS-D patients, propionate concentration was positively correlated with rapid colonic transit (57).

Beyond SCFA metabolomics, other metabolites—such as lipopolysaccharide (LPS), histamine, serotonin, tryptophan, and advanced glycosylation end-products—have been investigated (58–60). High FODMAP diet induces microbiota dysbiosis and elevate fecal LPS levels, triggering intestinal inflammation, characterized by upregulated mucosal expression of interleukin (IL) 1β, IL6, IL17, tumor necrosis factor (TNF)-*α*, and interferon (IF)-*γ*, which exacerbates visceral hypersensitivity. Conversely, low FODMAP diet reduces fecal LPS levels and intestinal inflammation, thereby improving visceral hypersensitivity (61). Our previous research in a stress-induced IBS mouse model revealed that fructo-oligosaccharides (FOS) aggravated visceral hypersensitivity and intestinal inflammation, with elevated IL23 expression in ileum, IL1β expression in colon, and increased mast cell counts in both regions (62). Clinical studies reported that low FODMAP intervention alleviates abdominal pain in IBS patients, correlating with reduced IL-6 and IL-10 and urinary histamine levels (37, 63). Germ-free mice colonized with fecal microbiota from IBS patients with high urinary histamine exhibited visceral hypersensitivity and mast cell activation; these effects were reversed by low-FODMAP feeding (59). Further *in vitro* studies identified *Klebsiella pneumoniae* (carrying histidine decarboxylase variants) as a key histamine producer, suggesting that histamine-secreting bacteria in IBS microbiota activate mast cells, which release neural mediators to regulate hypersensitivity (59). Tuck et al. demonstrated that pre low FODMAP intervention, fecal supernatant from IBS patients enhanced neuronal excitability and mechanical sensitivity of nociceptive afferent axons, while post-intervention supernatant suppressed these effects—a response mimicked by histamine receptor antagonists or protease inhibitors (64). Additionally, oral lactose or FOS administration increased visceral sensitivity in mice via mast cell accumulation and advanced glycosylation end-product receptor expression, preventable by the antiglycation agent pyridoxamine (60). These findings suggest that mast cell activation and glycation reactions are key mechanisms through which FODMAP diet exacerbate abdominal pain in IBS patients.

### 5.4 Regulating intestinal barrier function

Intestinal barrier dysfunction is a key pathogenic mechanism in IBS. Recent studies have suggested that the low FODMAP diet can regulate gastrointestinal symptoms by improving the intestinal barrier (Supplementary Table 4). Clinical study reported that low FODMAP diet reduces biomarkers of intestinal barrier impairment in IBS patients—such as intestinal fatty-acid binding protein, diamine oxidase and zonulin levels—suggesting improved small intestinal permeability and mucosal integrity (63). Michele et al. found that a 12-week low FODMAP diet improved intestinal barrier function and mucosal integrity while alleviating gastrointestinal symptoms in IBS patients (65). Animal studies support that FOS disrupt the mucus barrier and increase permeability (66, 67). Further, high lactose and fructo-oligosaccharide intake promote colonic mucus barrier dysfunction via glycation and mast cell activation, leading to reduced mucus layer thickness covering the fecal pellet (68). In rat models, a high FODMAP diet reduced tight junction protein expression and increased serum FITC-dextran, confirming barrier impairment; these effects were reversed by a low FODMAP diet (61). Similarly, Singh et al. found that a high FODMAP diet triggers mast cell–mediated barrier loss via LPS, whereas a low FODMAP diet restores mucosal pathophysiology (69).

### 5.5 Improving gut-brain axis dysfunction: a bridge connecting the gut and mental health

The gut-brain axis serves as a critical bidirectional pathway between the central and enteric nervous systems, and its dysfunction contributes significantly to chronic IBS symptomatology (70). Alterations in central visceral processing in IBS patients—such as reduced perceptual thresholds to intestinal stimuli (71), and heightened activity in brain regions governing emotion and pain regulation (72) —underline this connection.

A low FODMAP diet may ameliorate psychological symptoms by alleviating gastrointestinal distress and enhancing quality of life (QOL). Earlier work by Ledochowski et al. suggested that high FODMAP intake (e.g., fructose/sorbitol) may adversely affect mood, whereas a low FODMAP diet could alleviate depressive symptoms (73). Eswaran et al. observed greater improvements in QOL, anxiety, and activity disability with a low FODMAP diet versus traditional IBS dietary advice (74). Similarly, short- and long-term (6-week and 6-month) adherence to the low FODMAP diet reduced anxiety/depression levels and improved QOL in prospective studies (75). Mechanistically, Laura et al. linked these benefits to enhanced intestinal barrier integrity, suppressed inflammatory markers (e.g., IL-6, IL-10, LPS), and corrected microbial dysbiosis (63). Furthermore, fructan challenges in IBS patients evoke aberrant brain responses in pain-related regions (e.g., cerebellum, supramarginal gyrus, insula and thalamus), correlating with symptom severity (76). These findings imply that FODMAP-triggered symptoms in IBS are mediated through gut-brain axis dysregulation (Supplementary Table 5).

## 6 Conclusion

As an established therapeutic strategy, the low FODMAP diet significantly alleviates symptoms in IBS patients and improves their quality of life. While its short-term efficacy has been well validated, large-sample studies remain essential to evaluate long-term safety, durability of effects, and efficacy across IBS subtypes. Future research should focus on developing subtype-specific dietary strategies (e.g., protocols for IBS-D and IBS-C), which would refine patient selection criteria and gradations of intervention intensity. Current applications of the low FODMAP diet face significant challenges, particularly in ensuring patient adherence to the restrictive regimen in clinical practice. Enhancing intervention efficacy requires integration of patient education, dietitian collaboration, and digital tools (e.g., AI-driven diet-tracking platforms). Its mechanisms encompass complex interactions involving intestinal gas production and osmotic pressure, alterations in microbial composition and its metabolites, intestinal immune activation, visceral hypersensitivity, barrier function restoration, and gut-brain axis signaling. Future mechanistic studies should move beyond unidimensional analysis and adopt multi-omics integration to develop precise predictive models, thereby optimizing therapeutic efficacy and expanding clinical applicability.

Future research could focus on the following directions: (1) Optimizing personalized dietary plans based on gut microbiome and host metabolic profiles; (2) Exploring synergistic effects of combining dietary interventions with complementary therapies (e.g., probiotics, prebiotics, cognitive education) to enhance and sustain long-term treatment outcomes; (3) Developing AI- or big-data-driven tools for diet-symptom association analysis to support precise clinical decision-making; (4) Improving diagnostic criteria to resolve ambiguities between IBS and other functional/intolerance-related disorders, by distinguishing whether symptom improvement under low FODMAP diet is attributable to IBS-specific mechanisms or secondary to underlying food intolerances. Guiding precision interventions, such as lactose restriction for lactose intolerance/malabsorption, gluten-free diet for celiac disease, and broader FODMAP restriction for IBS and overlapping disorders, would further refine therapeutic strategies. Ultimately, the widespread adoption of the low FODMAP diet must be integrated with standardized diagnostic and therapeutic protocols, interdisciplinary collaboration (e.g., between gastroenterology and nutrition departments), and whole-cycle patient management to truly achieve the transition from symptom relief to long-term disease management.
