# Supplementary material for: Advances in the mechanism of low FODMAP diet in the treatment of irritable bowel syndrome: a review
Source: Front Nutr. 2026 Mar 12;13:1719048. doi: 10.3389/fnut.2026.1719048 (PMC13017274; doi:10.3389/fnut.2026.1719048)
Supplement: Supplementary file 1 [file Table_1.docx]

**Supplemental Table 1 Studies investigating the effect of FODMAP (or FODMAP restriction) on Intestinal Gas Production and GI symptom**

| **Author** | **Study Design** | **Participants** | **Intervention** | **Outcome Measures** | **Key Findings** |  |
| --- | --- | --- | --- | --- | --- | --- |
| Murray K et al. (32) | Four-way randomized single-blind crossover trial | 16 healthy volunteers | 40g either fructose, inulin, or a mixture  of glucose+ 40 g fructose vs 40g glucose (control) | 1. Colonic gas volume (MRI-AUC) | 1. Inulin ↑colonic gas (33±20 vs 19±14 L·min, *P*<0.05) & breath H₂ (18,000±9,000 vs 3,009±3,000 ppm/min, *P*<0.0001) |  |
|  |  |  |  | 2. Breath H₂ (AUC) | 2. No symptom differences between interventions (all *P*>0.5), scores low (≈1.0–1.3) |  |
|  |  |  |  | 3. GI symptoms (4-point scale) |  |  |
| Yang J et al. (35) | Randomized double-blind three-way crossover trial | 60 healthy controls & 60 IBS-D patients | 10g/20g/40g lactose | 1. LM: HBT positive if peak H₂ ≥20 ppm over baseline | Gas-symptom correlation: H₂ excretion (peak/AUC) positively correlated with TSS (r=0.28-0.35, *P*<0.001) |  |
|  |  |  |  | 2.LI: TSS increase ≥1 point with LM |  |  |
| Zhu Y et al. (34) | Cross-sectional study | 277 IBS & 64 healthy controls | 20g lactose | 1. Breath H₂ (AUC) | 1. H₂ AUC predicted bloating (OR=2.19) & borborygmi (OR=12.37) |  |
|  |  |  |  | 2. Rectal sensitivity | 2. Visceral hypersensitivity predicted bloating (OR=6.61, *P*=0.005) |  |
| Ong DK et al., (36) | Randomized single-blind crossover trial | 15 healthy & 15 IBS patients | LFD vs HFD | 1. Breath H₂/CH₄ (AUC-14h) | 1. HFD ↑H₂ in both groups (IBS: 62±23 vs 242±79 ppm·14h, *P*<0.0001) |  |
|  |  |  |  | 2. GI symptoms (0–3 scale) | 2. HFD ↓CH₄ in healthy (*P*=0.043) but not IBS |  |
|  |  |  |  | 3. Composite IBS score | 3. HFD worsened all symptoms in IBS (composite score: 2 vs 6, *P*=0.002) but only ↑flatus in healthy |  |
| McIntosh K et al. (37) | Randomized single-blind parallel trial | 40 IBS patients | LFD vs HFD | 1. Breath H₂ (AUC) | 1. Symptoms: ↓IBS-SSS in LFD group (289.6→208.0, *P*=0.0002), 72% responders vs 21% in HFD (*P*<0.009) |  |
|  |  |  |  | 2. IBS-SSS (symptom severity) | 2. Breath H₂: Minor ↓in LFD vs HFD (*P*<0.05) |  |
| Schindler V et al. (38) | Retrospective analysis | 110 DGBI patients | LFD after NCT (30g lactulose + 400mL liquid meal) | Breath H₂ during NCT | Higher hydrogen increased during proximal intestinal transit predicted better LFD response (P=0.045) |  |
|  |  |  |  |  |  |  |
| Somvanapanich P et al. (39) | Prospective study | 38 FGID patients | LFD | 1.Breath H₂/CH₄ levels | 1. Baseline H₂ >8 ppm predicted response (AUC=0.692, P<0.05) |  |
|  |  |  |  | 2.Bloating score | 2. 55% responders showed significant bloating improvement |  |
|  |  |  |  |  | 3. Responders had higher baseline H₂ (9.5 vs 4.5 ppm, P<0.05) and decreased post-diet |  |
| Ghoshal U.C. et al. (40) | Prospective experimental pilot study | 40 IBS patients & 20 healthy controls | LFD vs HFD | 1. Breath H₂ | 1. HFD meal ↑H₂ production vs LFD (greater in IBS patients) |  |
|  |  |  |  | 2. Symptom occurrence | 2. FMCT (H₂ rise >10 ppm + symptoms) predicted LFD response: sensitivity 78.6%, specificity 66.6%, accuracy 75.6% |  |
|  |  |  |  | 3. IBS-SSS change | 3. FMCT-positive patients had better LFD compliance and response (88% vs 50%, P=0.04) |  |
| Major G et al. (33) | Randomized, double-blind, three-period crossover trial | 29 IBS patients and 29 healthy volunteers | 40g Glucose 40g Fructose 40g Inulin | Symptom intensity (DCSS) Breath hydrogen concentration Colonic gas & volume (via MRI) | 1.Symptom threshold (DCSS ≥3) met more frequently after fructose (38%) and inulin (45%) than glucose (21%) in IBS patients.2. Symptom AUC was significantly higher in IBS patients than healthy volunteers.3. Fructose & Inulin produced significantly higher breath hydrogen AUC than glucose in both groups (P < 0.05).4. Inulin led to higher colonic gas AUC than glucose (P < 0.05).5. Fructose & Inulin resulted in higher colonic volume AUC than glucose. |  |
| Wu et al. (76) | Randomized, double-blind, placebo-controlled crossover trial | 13 female IBS patients vs. 13 healthy controls | Intragastric infusion of fructans (40g) vs. glucose (40g) vs. saline (500 mL) | GI symptom ratings (VAS), Abdominal MRI (small bowel motility, colon gas/volume) | Fructans induced significantly more cramps, pain, and nausea in IBS patients vs. controls, despite similar gut physiological responses (motility, gas, volume). |  |

Low FODMAP diet (LFD); High FODMAP diet (HFD); FODMAP meal challenge test (FMCT); Lactose malabsorption (LM); Lactose intolerance (LI);Total symptom score (TSS); Hydrogen breath test (HBT); nutrient challenge test (NCT)

**Supplemental Table 2 Studies investigating the effect of FODMAP (or FODMAP restriction) on Intestinal Content Osmolarity**

| **Author** | **Study Design** | **Participants** | **Intervention** | **Outcome Measures** | **Key Findings** |
| --- | --- | --- | --- | --- | --- |
| Murray K et al. (32) | Randomized single-blind crossover trial | 16 healthy volunteers | 40 g glucose  40 g fructose  40 g inulin  40 g fructose+40g glucose | SBWC: AUC (0-5h) quantified by MRI | Greater SBWC fructose (67mL/min) versus glucose(36 L/min; p<0.005). No SBWC difference inulin vs glucose or fructose + glucose vs fructose |
|  |  |  |  |  |  |
|  |  |  |  |  |  |
|  |  |  |  |  |  |
| Barrett JS et al. (41) | Randomized single-blinded crossover trial | 12 ileostomates without small intestinal disease (10 analyzed) | HFD vs.LFD | Ileal effluent weight (g), water content (mL), dry weight (g), FODMAP recovery rate measured by HPLC | HFD increased effluent weight by 22 (95% CI: 5–39%), water content by 20% (2–38%), and dry weight by 24% (4–43%). 32% of ingested FODMAPs recovered in effluent, confirming osmotic activity and increased fluid delivery. |
| Marciani L et al. (42) | Randomized single-blinded crossover trial | 11 healthy volunteers | 17.5 g mannitol vs. 17.5 g glucose | SBWC quantified by MRI at 30-min intervals for 3.5 hours | Mannitol meal increased SBWC to 381 mL (IQR: 343–491 mL) vs. 47 mL (18–78 mL) with glucose at 40 min (P < 0.001). Mannitol stimulated net secretion while glucose promoted absorption. |
| Major G et al. (33) | Randomized double blind crossover trial | 29 IBS patients and 29 healthy volunteers | 40 g of glucose, fructose, or inulin | SBWC measured by MRI | SBWC: AUC was higher after fructose than glucose (P < 0.05), but no significant difference in SBWC between inulin and glucose |
|  |  |  |  |  |  |
|  |  |  |  |  |  |
|  |  |  |  |  |  |

Small Bowel Water Content (SBWC)

**Supplemental Table 3 Studies investigating the effect of FODMAP (or FODMAP restriction) on immune activation: From " Microbiomics" to "Metabolomics"**

| **Author** | **Study Design** | **Participants / Model** | **Duration (weeks)** | **Method (Microbiota)** | **Findings**  **(Microbiota)** | **Method (Metabolite)** | **Findings**  **(Metabolite）** | **Method (immune activation)** | **Findings**  **(immune activation)** |
| --- | --- | --- | --- | --- | --- | --- | --- | --- | --- |
| McIntosh et al.(37) | Randomized, single-blind, parallel-group controlled trial | 40 IBS patients, 37 completed (19 LFD, 18 HFD) | 3 | 16S rRNA gene profiling (Illumina) | LFD (↑Actinobacteria richness/diversity). HFD (↓bacteria involved in gas consumption) | GC-MS on urine | Urinary histamine reduced eight folds in LFD group. | - | Urinary histamine reduction suggests modulation of immune activation. |
| Vervier et al. (43) | Prospective single-center case-control study | 56 IBS patients & household controls (41 pairs completed) | 4 | Shotgun metagenomic sequencing | Pathogenic-like subgroup (↑Firmicutes, ↓Bacteroidetes) shifted towards a healthier profile post-LFD. | - | - | - | - |
| Zhang et al. (44) | Parallel-group randomized controlled trial | 108 IBS-D patients (100 completed) | 3 | 16S rRNA sequencing, Metabolomics | LFD reduced carbohydrate-fermenting bacteria (e.g., Bifidobacterium, Bacteroides). | Metabolomics (SCFA) | LFD decreased glycolytic fermentation activity.Patients with higher baseline saccharolytic capacity had a more severe symptom burden and a better response to the LFD. | - | - |
| Chumpitazi et al. (45) | Double-blind, randomized, crossover trial | 33 children with Rome | 1 (per diet, with 5-day washout) | 16S rRNA sequencing, PICRUSt (functional prediction) | Responders at baseline had ↑glycolytic capacity bacteria (e.g., Bacteroidaceae, Ruminococcaceae). | - | - | - | - |
| Conley et al. (46) | Prospective dietary intervention study (paired with controls) | 56 IBS patients & household controls | 4 | - | - | Metagenomic sequencing; GC-MS | Responders (IBS-P) had baseline enrichment of SCFA fermentation features; LFD significantly reduced SCFA production. | - | - |
| Wilson et al. (47) | Blinded, randomized controlled trial | 69 IBS patients | 4 | - | - | GC-MS; GC | Elevated baseline fecal propionate and cyclohexanecarboxylic acid predicted clinical responders. | - | - |
| Ameen et al. (48) | Intervention study (integrated data analysis) | IBS patients | - | Shotgun metagenomics | Responders had ↑abundance of methane and SCFA metabolic pathways at baseline. | - | - | - | - |
| So et al. (49) | Systematic review | IBS patients and controls | 3-4 (one trial 12) | 16S rRNA sequencing, FISH, qPCR | Consistent reduction in Bifidobacteria; no clear effects on overall diversity, other specific taxa, SCFAs, or fecal pH. | GC, GLC | No difference in total or specific fecal SCFA concentrations between LFD and controls. | - | - |
| De Palma et al. (59) | Human-to-mouse FMT & in vitro study | IBS patients & germ-free mice | 3 | Bacterial culture; 16S rRNA sequencing | Identified Klebsiella pneumoniae as a key histamine producer. | LC-MS/MS; ELISA | Increased luminal and urinary histamine in high-histamine IBS microbiota groups; no difference in SCFAs. | Visceral sensitivity testing; Immunohistochemistry | Microbiota from high-histamine IBS patients induced visceral hypersensitivity and mast cell activation, reversible by LFD. |
| Kamphuis et al. (60) | Animal interventional study | C57BL/6 mice gavaged with lactose or fed a FOS diet | 3 (lactose) / 6 (FOS) | 16S rRNA gene sequencing | No significant alterations in fecal microbiota composition. | LC-MS | Increased levels of reactive carbonyl compounds (glycation agents) in fecal samples. | Immunofluorescence (mast cells, AGER) | Increased visceral sensitivity, colonic mast cell density, and AGER expression in the colon epithelium; prevented by pyridoxamine. |
| Zhou et al. (61) | Animal interventional study and human pilot study | Mice (C57BL/6), IBS-D patients and healthy subjects | 2 (rats) / 4 (patients) | 16S rRNA sequencing | HFM diet increased Gram-negative bacteria (e.g., Akkermansia muciniphila). | ELISA; GC | HFD diet increased fecal and serum LPS; increased SCFA production in rats. | qPCR (cytokines); Visceral sensitivity testing | HFD ↑pro-inflammatory cytokines (IL-1β, IL-6, IL-17, TNF-α, IFN-γ) and visceral hypersensitivity; mediated by LPS-TLR4 signaling. |
| Chen et al. (62) | Animal interventional study | C57BL/6 mice (Water Avoidance Stress model) | 2 | - | - | - | - | qPCR; Immunohistochemistry | Fructo-oligosaccharides (FOS) ↑IL-23 (ileum), ↑IL-1β (colon), and ↑mast cell counts. |
| Prospero et al. (63) | Non-randomized, unblinded, single-arm intervention study | 20 IBS-D patients | 12 | - | - | ELISA ; Colorimetric assay/HPLC (urinary indican/skatole) | Significant decrease in serum LPS; urinary indican remained elevated (NS); skatole decreased significantly (within normal range). | ELISA (cytokines) | decreased IL-6 and IL-10; no significant change in IL-8, TNF-α. |
| Tuck et al. (64) | Human interventional study | IBS patients | 3 | - | - | Neuronal patch-clamp assay | Post-LFD fecal supernatants suppressed neuronal excitability. | Neuronal patch-clamp assay | Pre-LFD fecal supernatants enhanced neuronal excitability; effects mimicked by histamine receptor antagonists/protease inhibitors. |

Phylogenetic Investigation of Communities by Reconstruction of Unobserved States (PICRUSt)

**Supplemental Table 4 Studies investigating the effect of FODMAP (or FODMAP restriction) on Intestinal Barrier Function**

| **Author** | **Study Design** | **Participants / Model** | **Duration (weeks)** | **Method** | **Findings** |
| --- | --- | --- | --- | --- | --- |
| Zhou et al. (61) | Animal: Controlled interventional; Human: Non-randomized, unblinded, single-arm pilot | Animal: Adult male Wistar rats; Human: 6 IBS-D patients & healthy subjects | 2 (Animal) / 4 (Human) | Animal: TEER, serum FITC-dextran, qPCR/Western blot (tight junction proteins) | A HFD in rats induced intestinal barrier dysfunction (reduced TEER, increased serum FITC-dextran, decreased ZO-1/occludin).These effects were reversed by a low FODMAP diet. |
| Prospero et al. (63) | Non-randomized, unblinded, single-arm intervention | 20 IBS-D patients | 12 | ELISA (I-FABP, DAO, zonulin); Sugar absorption test | LFD reduced biomarkers of intestinal barrier impairment (I-FABP, DAO, zonulin) and improved intestinal permeability (lactulose/mannitol ratio). |
| Linsalata et al. (65) | Prospective interventional study | 38 IBS-D patients | 1 | Sugar absorption test; fecal/serum zonulin; erythrocyte membrane PUFA analysis | LFD improved intestinal permeability (reduced lactulose/mannitol ratio), decreased fecal zonulin, and modulated erythrocyte membrane PUFA composition (increased n-3, decreased n-6/n-3 ratio). |
| Genda et al. (66) | Interventional animal study | Male Wistar rats | 9 days or 58 days | Gut permeability assays (chromium-EDTA); immunohistochemistry | Short-term FOS ingestion increased cecal IgA, gut permeability, and mucosal inflammation; effects attenuated after long-term ingestion. Associated with mucus layer disruption. |
| Ten Bruggencate et al. (67) | Interventional animal study | Male Wistar rats | 2 (pre-infection adaptation) | Intestinal permeability (chromium-EDTA); mucin analysis | FOS increased intestinal permeability, mucin secretion, and bacterial translocation, indicating barrier impairment. |
| Kamphuis et al. (68) | Interventional animal study | Adult male C57BL/6 mice | 3 | Histology (mucus layer thickness); Immunofluorescence (mast cells, CML) | High lactose/FOS intake impaired colonic mucus barrier (reduced thickness, increased variability), increased discharging goblet cells, mucosal mast cell counts, and epithelial AGE (CML) levels. Pyridoxamine co-treatment prevented these effects. |
| Singh et al. (69) | Animal: Randomized controlled; Human: Non-randomized, unblinded, single-arm intervention | Animal: WT and mast cell-deficient (Kit^W/W–v^) mice; Human: 6 IBS-D patients | 2 (Animal) / 4 (Human) | Animal: TEER, FITC-dextran flux, qPCR/Western blot (tight junction proteins) | A HFD induced colonic barrier loss (reduced TEER, increased permeability, decreased ZO-1/Occludin). A lFD in IBS-D patients improved barrier function (increased JAM-A/ZO-1 expression) |

**Supplemental Table 5 Studies investigating the effect of FODMAP (or FODMAP restriction) on Intestinal Barrier Function**

| **Author** | **Study Design** | **Participants** | **Intervention** | **Outcome Measures** | **Key Findings** |
| --- | --- | --- | --- | --- | --- |
| Prospero et al. (63) | Non-randomized, unblinded, single-arm intervention | 20 IBS-D patients | LFD for 12 weeks | Psychological: SCL-90-R (anxiety, somatization, psychoticism), QoL questionnaires (IBS-QoL, SF-36); ELISA (barrier markers, cytokines); LPS assay | LFD improved psychological state (anxiety, somatization, psychoticism) and QOL, which was linked to enhanced intestinal barrier integrity, reduced inflammatory markers (IL-6, IL-10), and lower LPS levels. |
| Ledochowski et al.(73) | Prospective dietary intervention (non-randomized, unblinded, single-arm) | 53 adults with fructose malabsorption | 4-week fructose- and sorbitol-restricted diet | Beck’s Depression Inventory (BDI); symptom scales for meteorism, stool frequency, and well-being | Significant reduction in depressive symptoms (65.2% BDI decrease, p<0.0001), meteorism (p<0.0001), and stool frequency (p<0.01). Supports gut-brain axis modulation via potential improvement in tryptophan availability. |
| Eswaran et al. (74) | Randomized controlled trial (single-blind) | 84 IBS-D patients | LFD vs. mNICE diet for 4 weeks | IBS-QOL, HADS (anxiety/depression), Work Productivity and Activity Impairment | LFD led to significantly greater improvements in IBS-specific quality of life, anxiety scores, and activity impairment compared to the mNICE diet. |
| Kortlever et al. (75) | Prospective observational study (non-randomized) | 101 IBS patients (56 completed) | Low FODMAP diet for 6 weeks, with follow-up at 6 weeks and 6 months | IBS-QOL, GSRS (GI symptoms), HADS, Fatigue Impact Scale, Subjective Vitality Scale, Happiness Measures | The LFD was associated with significant long-term improvements in quality of life, GI symptoms, fatigue, anxiety, and depression, and increased happiness and vitality. Improvements in psychological state correlated with GI symptom reduction. |
| Wu et al. (76) | Randomized, double-blind, placebo-controlled crossover trial | 13 female IBS patients vs. 13 healthy controls | Intragastric infusion of fructans (40g) vs. glucose (40g) vs. saline (500 mL) | GI symptom ratings (VAS), fMRI brain activity | Altered brain responses to fructans in pain-related regions (e.g., insula, cingulate cortex, thalamus) were observed in IBS and correlated with symptom severity, indicating gut-brain axis dysregulation. |

Modified diet recommended by the National Institute for Health and Care; Excellence (mNICE); Visual analogue scale(VAS)
